# A simple nomogram for predicting the mortality of PICU patients with sepsis-associated encephalopathy: a multicenter retrospective study

**DOI:** 10.3389/fneur.2024.1418405

**Published:** 2024-07-29

**Authors:** Guan Wang, Yan Gao, Yanan Fu, Qin Huo, Enyu Guo, Qin Jiang, Jing Liu, Xinzhu Jiang, Xinjie Liu

**Affiliations:** ^1^Department of Pediatrics, Qilu Hospital of Shandong University, Jinan, Shandong, China; ^2^Qilu Hospital of Shandong University, Jinan, Shandong, China; ^3^Department of Medical Engineering, Qilu Hospital of Shandong University, Jinan, Shandong, China; ^4^Department of General Medicine, The Fourth People's Hospital of Jinan, Jinan, Shandong, China; ^5^Department of Pediatrics, Jining First People's Hospital, Jining, Shandong, China; ^6^Department of Pediatrics, Jinan Children’s Hospital of Shandong University, Jinan, Shandong, China; ^7^Department of Biostatistics, School of Public Health, Cheeloo Cholege of Medicine, Shandong University, Jinan, Shandong, China

**Keywords:** sepsis-associated encephalopathy, nomogram, prediction, mortality, PICU

## Abstract

**Background:**

As one of the serious complications of sepsis in children, sepsis-associated encephalopathy (SAE) is associated with significantly poor prognosis and increased mortality. However, predictors of outcomes for pediatric SAE patients have yet to be identified. The aim of this study was to develop nomograms to predict the 14-day and 90-day mortality of children with SAE, providing early warning to take effective measures to improve prognosis and reduce mortality.

**Methods:**

In this multicenter, retrospective study, we screened 291 patients with SAE admitted to the PICU between January 2017 and September 2022 in Shandong Province. A least absolute shrinkage and selector operation (LASSO) method was used to identify the optimal prognostic factors predicting the outcomes in pediatric patients with SAE. Then, multivariable logistic regression analysis was performed based on these variables, and two nomograms were built for visualization. We used the area under the curve (AUC), calibration curves and decision curves to test the accuracy and discrimination of the nomograms in predicting outcomes.

**Results:**

There were 129 patients with SAE in the training cohort, and there were 103 and 59 patients in the two independent validation cohorts, respectively. Vasopressor use, procalcitonin (PCT), lactate and pediatric critical illness score (PCIS) were independent predictive factors for 14-day mortality, and vasopressor use, PCT, lactate, PCIS and albumin were independent predictive factors for 90-day mortality. Based on the variables, we generated two nomograms for the early identification of 14-day mortality (AUC 0.853, 95% CI 0.787–0.919, sensitivity 72.4%, specificity 84.5%) and 90-day mortality (AUC 0.857, 95% CI 0.792–0.923, sensitivity 72.3%, specificity 90.6%), respectively. The calibration plots for nomograms showed excellent agreement of mortality probabilities between the observed and predicted values in both training and validation cohorts. Decision curve analyses (DCA) indicated that nomograms conferred high clinical net benefit.

**Conclusion:**

The nomograms in this study revealed optimal prognostic factors for the mortality of pediatric patients with SAE, and individualized quantitative risk evaluation by the models would be practical for treatment management.

## 1 Introduction

Severe sepsis, normally treated in the pediatric intensive care unit (PICU), is a leading threat to the life and health of children worldwide (1). Sepsis-associated encephalopathy (SAE) is defined as a diffuse brain disorder without direct central nervous system (CNS) infection and is one of the most critical complications in children with sepsis (2, 3). Previous studies have indicated that in all sepsis patients, the incidence of SAE varies from 8% to more than 70% (4). Importantly, SAE can cause a dramatically poorer prognosis, and the mortality rate of sepsis patients significantly increases with increasing SAE severity to a maximum of 70% (5). Furthermore, surviving patients with SAE are likely to develop permanent neurocognitive impairment, which can significantly reduce the quality of life and bring great burden to their families and even society (5–7). Based on these findings, early identification and medical intervention for SAE is very important to improve the prognosis and reduce the mortality of patients with sepsis, especially children.

Li et al. proposed an accurate discriminative model and a simplified scoring model, which are applicable tools for assessing the severity in pediatric sepsis patients (8). However, the risk factors for predicting the mortality of SAE patients, especially pediatric patients, remain incompletely understood to date. Huang et al. (9) proposed that the hemoglobin-to-red cell distribution width ratio (HRR) was associated with the prognosis and mortality of SAE, but the limitation was that there was no long-term out-of-hospital follow-up. Peng et al. (10) developed machine learning (ML) models to predict the risk of 30-day mortality of SAE, but external validation was lacking. In addition, few high-quality studies have focused on mortality prediction of SAE in children.

The nomogram model is a powerful and practical decision-making tool that has been proven to provide highly accurate risk assessment in individual patients (11). In the current study, the main objective was to develop nomograms based on routine variables measured easily within a few hours of PICU admission to help clinicians identify children with SAE at high risk of death.

## 2 Materials and methods

### 2.1 Study design

This retrospective, multicenter observational study was performed in three hospitals designated for PICU patients with SAE in Shandong Province. Among the three centers, Qilu Hospital of Shandong University with 28 PICU beds was included as the training cohort, while Jinan Children’s Hospital of Shandong University with 30 PICU beds and Jining First People’s Hospital with 27 PICU beds were included as external validation cohorts. The study was registered at the Chinese Clinical Trial Registry (ChiCTR2100048600) and approved by the institutional ethics review board of Qilu Hospital of Shandong University (KYLL-202202-027-1). Informed consent was not required because of the observational and retrospective nature of the study.

### 2.2 Participants

SAE is defined as sepsis combined with alterations in mental status, cognitive or behavioral abnormalities, and a Glasgow Coma Score (GCS) ≤14 during the patient’s stay in the PICU (2, 3). Cognitive and neuropsychiatric symptoms of SAE include lack of focus, confusion, agitation, heightened sensitivity, reduced psychomotor function, drowsiness, stupor, and coma. These symptoms were thoroughly recorded in the medical documentation by healthcare professionals. SAE patients were enrolled during the period from January 2017 to September 2022 in the training cohort and from January 2017 to May 2022 in the validation cohorts. We enrolled patients with the following inclusion criteria: (1) age between 28 days and 18 years; (2) a diagnosis of sepsis according to the Sepsis 3.0 definition (12); and (3) altered mental status with cognitive or behavioral abnormalities accompanied by GCS ≤ 14 during the PICU stay. The exclusion criteria were as follows: (1) primary cerebral disease, including brain trauma, cerebrovascular disease, epilepsy, intracranial infection and autoimmune encephalitis; (2) metabolic encephalopathy caused by diabetic ketoacidosis, hypoglycemia, hepatic encephalopathy and inherited metabolic diseases; and (3) missing laboratory data. Most of the SAE patients were diagnosed within 72 h after PICU admission, while others were diagnosed longer after PICU admission. The earliest time for SAE diagnosis was 8 h after PICU admission.

### 2.3 Data collection and study outcomes

Demographic and clinical data were retrieved from the electronic medical record systems of the three hospitals. Two researchers who were totally unaware of the study design were responsible for the data collection. Vital signs, the pediatric critical illness score (PCIS) and laboratory data were recorded during the first 24 h after PICU admission. If there were multiple measurement results within the first 24 h, we would choose the worst one. The PCIS system mainly includes heart rate, systolic blood pressure, respiration, arterial partial oxygen pressure, pH, and creatinine, etc. We collected data on the suspected infection site and microorganisms, the use of vasopressors (adrenaline, norepinephrine, dopamine, dobutamine and other vasopressors) and advanced life support (including mechanical ventilation and renal replacement therapy) during the PICU stay. The primary outcomes include death within 14 days and 90 days after PICU admission.

### 2.4 Statistical analysis

The normal distributions of variables were checked by Shapiro–Wilk tests. Continuous variables with normal distributions were tested by unpaired Student’s *t* test and are presented as the mean ± standard deviation (SD), whereas nonnormally distributed continuous variables were tested by the Mann–Whitney U-test and are presented as the median (interquartile range, IQR). Categorical variables were tested by chi-square analysis or Fisher’s exact test accordingly and described as frequencies (proportions). To identify the relative importance of the variables and build prediction models, a least absolute shrinkage and selection operator (LASSO) algorithm was tested in the training cohort. Then, we performed a multivariable logistic analysis based on the LASSO regression model, presenting odds ratios (ORs) and 95% confidence intervals (CIs). The selected variables were used to build the nomogram and online calculator to predict the death risk of SAE patients in the PICU. We used the area under the curve (AUC) to quantitatively evaluate the discriminative performance of the nomogram. Calibration curves were plotted by bootstraps of 1,000 resamples to assess the association between the observed incidence and the predicted probability. The clinical usefulness of the nomogram was determined by decision curve analysis (DCA) in training and validation sets, which quantified the net benefits at various threshold probabilities. Statistical analyses were conducted using R version 3.4.3 (The R Foundation for Statistical Computing, Vienna, Austria).^1^ Statistical significance was defined as a two-sided *p*-value of <0.05.

## 3 Results

### 3.1 Demographic and clinical characteristics

**Figure 1:**
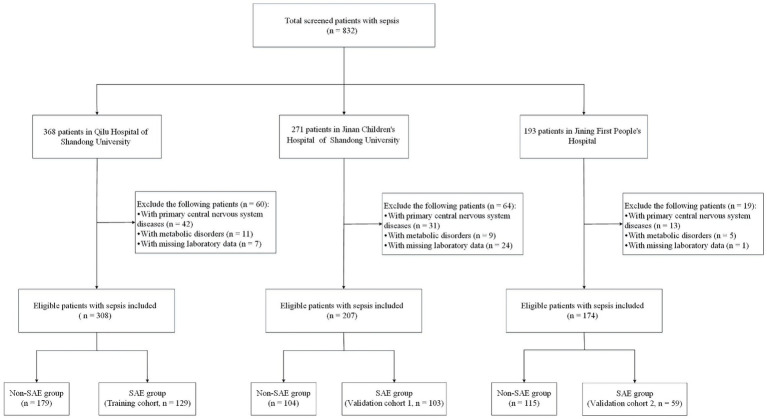
Flowchart of study participants in the training and validation groups. SAE, sepsis-associated encephalopathy.

The selection of the study cohort is illustrated in Figure 1. A total of 291 patients were enrolled in our study, including 129 patients at Qilu Hospital of Shandong University (training cohort), 103 patients at Jinan Children’s Hospital of Shandong University (validation cohort 1), and 59 patients at Jining First People’s Hospital (validation cohort 2). The baseline and clinical characteristics of the training and validation cohorts are shown in Table 1. The training cohort consisted of 79 (61.24%) males and 50 (38.76%) females with a median age of 51 months (IQR 13–137 months). The 14-day and 90-day mortality rates in the training cohort were 44.96 and 50.39%, respectively. Validation cohort 1 comprised 52 (50.49%) males and 51 (49.51%) females with a median age of 48 months (IQR 10.5–92.5 months), while validation cohort 2 comprised 38 (64.41%) males and 21 (35.59%) females with a median age of 42 months (IQR 10.5–80.5 months). The baseline and clinical characteristics were not significantly different between the training cohort and validation cohorts (Table 1).

**Table 1:** Baseline and clinical characteristics of the training and validation cohorts.

Characteristics	Training cohort (*n* = 129)	Validation cohort 1 (*n* = 103)	Validation cohort 2 (*n* = 59)	*p* value
Age (month)	51 (13, 137)	48 (10.5, 92.5)	42 (10.5, 80.5)	0.134
Gender (male, %)	79 (61.24)	52 (50.49)	38 (64.41)	0.14
Vital signs				
HR (beats/min)	128 (105, 146)	132 (100.5, 161.5)	125 (101.5, 153)	0.662
RR (breaths/min)	30 (23, 38)	32 (23, 39)	29 (25, 33)	0.396
SBP (mmHg)	103 (88, 116)	98 (85, 107.5)	99 (87.5, 120)	0.064
DBP (mmHg)	60 (48, 68)	55 (45.5, 67)	54 (43.5, 65)	0.377
Temperature > 38°C, *n* (%)	60 (46.51)	41 (39.81)	23 (38.98)	0.484
Vasopressor use within 24 h after PICU admission, *n* (%)	45 (34.88)	46 (44.66)	28 (47.46)	0.167
14-day mortality, *n* (%)	58 (44.96)	45 (43.69)	22 (37.29)	0.604
90-day mortality, *n* (%)	65 (50.39)	62 (60.19)	29 (49.15)	0.246

SAE, sepsis-associated encephalopathy; HR, heart rate; RR, respiratory rate; SBP, systolic pressure; DBP, diastolic pressure; PICU, pediatric intensive care unit.

The baseline and clinical characteristics of SAE patients in the training cohort are shown in Table 2. There were 65 nonsurvivors with a median age of 74 months (IQR 15–138 months). No significant differences were found in age or sex between survivors and nonsurvivors (*p* > 0.05). The respiratory rate of nonsurvivors was significantly higher than that of survivors (32 with IQR 25–40 breaths/min vs. 27 with IQR 22–33.25 breaths/min, *p* = 0.015). For both nonsurvivors and survivors, respiratory infection occupied the highest proportion among suspected infection foci. Interestingly, blood infection seemed to be more common in the nonsurvivors, but the difference did not reach statistical significance (18.46% vs. 14.06%, *p* > 0.05). In addition, gram-negative bacteria were the most common microorganisms in the nonsurvivors, while gram-positive bacteria were the most common microorganisms in the survivors. Nonsurvivors tended to have more vasopressor use within 24 h after PICU admission (47.69% vs. 21.88%, *p* = 0.004) and more mechanical ventilation therapy during the PICU stay (60% vs. 12.5%, *p* < 0.001). Moreover, nonsurvivors were more likely to experience septic shock, and they had longer hospital and PICU stay times (*p* < 0.05). Furthermore, the levels of alanine transaminase (ALT), total bilirubin (TBIL), creatine kinase-MB (CK-MB), lactic dehydrogenase (LDH), international normalized ratio (INR), prothrombin time (PT), D-dimer, brain natriuretic peptide (BNP), procalcitonin (PCT) and lactate were significantly higher in nonsurvivors (*p* < 0.05). The levels of platelets (PLT), hemoglobin (HB), albumin, fibrinogen (Fib) and the pediatric critical illness score (PCIS) were significantly lower in nonsurvivors (*p* < 0.05).

**Table 2:** Baseline and clinical characteristics of the survivors and nonsurvivors in the training cohort.

Characteristics	Survivors (*n* = 64)	Nonsurvivors (*n* = 65)	*P* value
Age (month)	47.5 (11.75, 128.75)	74 (15, 138)	0.411
Gender (male, %)	35 (54.69)	44 (67.69)	0.182
Vital signs			
HR (beats/min)	122.36 ± 23.64	131.72 ± 29.97	0.051
RR (breaths/min)	27 (22, 33.25)	32 (25, 40)	0.015
SBP (mmHg)	104.48 ± 20.13	101.65 ± 20.9	0.434
DBP (mmHg)	59.88 ± 13.93	56.69 ± 14.3	0.203
Temperature > 38°C, *n* (%)	25 (39.06)	35 (53.85)	0.132
Suspected infection focus, *n* (%)			0.935
Respiratory infection	32 (50)	33 (50.77)	
Blood infection	9 (14.06)	12 (18.46)	
Urinary tract infection	1 (1.56)	1 (1.54)	
Intra-abdominal infection	3 (4.69)	2 (3.08)	
Other or unknown	19 (29.69)	17 (26.15)	
Blood culture-positive, *n* (%)	9 (14.06)	12 (18.46)	0.661
Microorganisms, *n* (%)	20 (31.25)	24 (36.92)	0.621
Gram-positive	10 (15.62)	4 (6.15)	0.148
Gram-negative	6 (9.38)	13 (20)	0.146
Fungus	2 (3.12)	4 (6.15)	0.680
Multiple infection (two or more)	4 (6.25)	7 (10.77)	0.546
Vasopressor use within 24 h after PICU admission, *n* (%)	14 (21.88)	31 (47.69)	0.004
Advanced life support during PICU stay time			
Mechanical ventilation, *n* (%)	8 (12.5)	39 (60)	< 0.001
Renal replacement therapy, *n* (%)	6 (9.38)	10 (15.38)	0.442
Outcome			
Septic shock, *n* (%)	5 (7.81)	18 (27.69)	< 0.001
Hospital stay time (days)	11 (8, 21)	9 (4, 18)	0.025
PICU stay time (days)	9 (6.75, 16)	5 (2, 14)	0.008
WBC (×10^9^/L)	10.19 (7.22, 15.76)	7.84 (2.04, 21.59)	0.317
PLT (×10^9^/L)	311.5 (179, 477.25)	218 (86, 380)	0.015
HB (g/L)	105.77 ± 25.8	93.71 ± 27.51	0.011
ALT (U/L)	21 (11.75, 40.25)	37 (21, 69)	0.005
TBIL (umol/L)	6.9 (4.47, 10.53)	10.7 (5.9, 22.9)	0.004
Albumin (g/L)	37.46 ± 6.39	31.46 ± 7.74	< 0.001
CK-MB (mg/L)	1.95 (1.3, 4.18)	4 (2, 12)	0.007
Scr (umol/L)	29.5 (22, 39.25)	34 (23, 53)	0.121
BUN (mmol/L)	3.6 (2.6, 5.8)	4.6 (3, 7.7)	0.069
LDH (U/L)	374 (279.75, 526.5)	585 (368, 1756)	< 0.001
Na (mmol/L)	137 (134.75, 139)	135 (131, 139)	0.081
K (mmol/L)	4.3 ± 0.61	4.09 ± 0.76	0.084
Fib (g/L)	3.1 (2.47, 4.12)	1.98 (1.25, 3.71)	0.011
INR	1.19 (1.04, 1.29)	1.22 (1.1, 1.42)	0.049
PT (s)	13.6 (12.9, 15.22)	14.5 (13.6, 17.8)	< 0.001
APTT (s)	37.65 (34.6, 41.55)	38.6 (34.8, 44)	0.238
D-dimer (ug/mL)	1.48 (0.6, 3.94)	2.57 (1.07, 15.16)	0.007
BNP (ng/L)	189.5 (82.5, 361.25)	503 (217, 1,390)	< 0.001
CRP (mg/L)	37.45 (8.33, 93.65)	50.3 (5.04, 126.44)	0.298
PCT (ug/L)	0.26 (0.12, 0.46)	0.52 (0.24, 5.56)	< 0.001
Lactate (mmol/L)	1.55 (1, 2.22)	2.6 (1.6, 4.3)	< 0.001
PH	7.42 (7.36, 7.46)	7.41 (7.32, 7.46)	0.901
PaO_2_ (mmHg)	82 (70.25, 94.5)	81 (52, 100)	0.88
PaCO_2_ (mmHg)	36 (30, 42)	35 (31, 41)	0.897
SaO_2_ (%)	98.05 (96, 98.93)	97.9 (93.3, 98.9)	0.287
Glucose (mmol/L)	6.2 (5.3, 8.2)	6.5 (5, 8.9)	0.878
PCIS	93 (88, 96)	84 (76, 90)	< 0.001

WBC, white blood cell; PLT, platelet; HB, hemoglobin; ALT, alanine transaminase; TBIL, total bilirubin; CK-MB, creatine kinase-MB; Scr, serum creatinine; BUN, blood urea nitrogen; LDH, lactic dehydrogenase; Na, serum sodium; K, serum potassium; Fib, fibrinogen; INR, international normalized ratio; PT, prothrombin time; APTT, activated partial thromboplastin time; BNP, brain natriuretic peptide; CRP, C reactive protein; PCT, procalcitonin; PaO2, arterial oxygen partial pressure; PaCO2, arterial partial pressure of carbon dioxide; SaO2, arterial oxygen saturation; PCIS, pediatric critical illness score.

### 3.2 Prognostic factors of SAE

**Figure 2:**
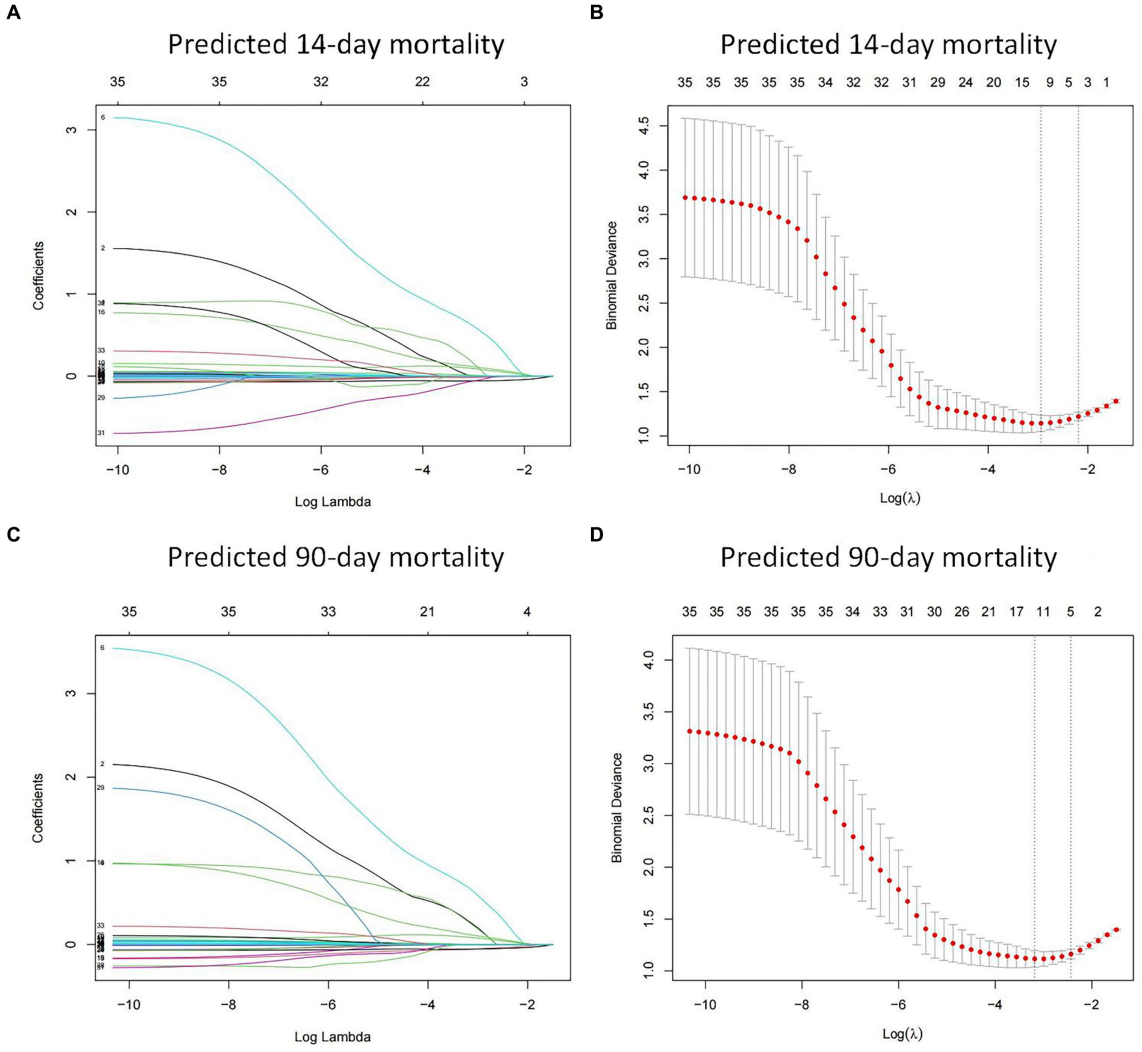
Feature selection using the least absolute shrinkage and selection operator (LASSO) regression in the training cohort. LASSO coefficient profiles of the 35 features for predicting 14-day mortality **(A)** and 90-day mortality **(C)**. Identification of the optimal penalization coefficient (λ) in the LASSO model was performed via 10-fold cross-validation for predicting 14-day mortality **(B)** and 90-day mortality **(D)**.

**Figure 3:**
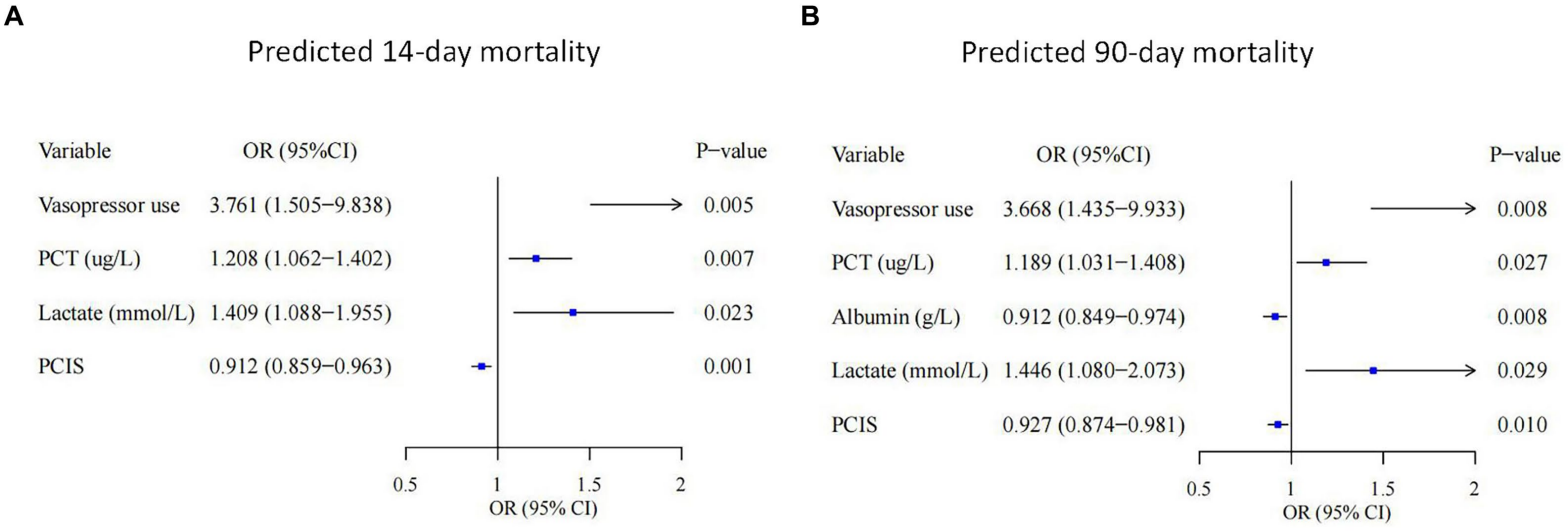
Forest plots showing the results of multivariate analyses in the 14-day mortality **(A)** and 90-day mortality **(B)** groups. PCT, procalcitonin; PCIS, pediatric critical illness score; OR, odds ratio; CI, confidence interval.

A total of 35 features were collected from each patient in the training cohort for LASSO regression analysis (Figure 2). The results showed that vasopressor use, PCT, lactate and PCIS were predictive factors for 14-day mortality, and vasopressor use, PCT, lactate, PCIS and albumin were predictive factors for 90-day mortality with maximal AUC. In multivariate analysis, vasopressor use (OR 3.761, 95% CI 1.505–9.838), PCT (OR 1.208, 95% CI 1.062–1.402), lactate (OR 1.409, 95% CI 1.088–1.955), and PCIS (OR 0.912, 95% CI 0.859–0.963) were independently associated with mortality at 14 days (Figure 3A), while vasopressor use (OR 3.668, 95% CI 1.435–9.933), PCT (OR 1.189, 95% CI 1.031–1.408), albumin (OR 0.912, 95% CI 0.849–0.974), lactate (OR 1.446, 95% CI 1.080–2.073), and PCIS (OR 0.927, 95% CI 0.874–0.981) were independently associated with mortality at 90 days (Figure 3B).

### 3.3 Construction and validation of the nomogram

**Figure 4:**
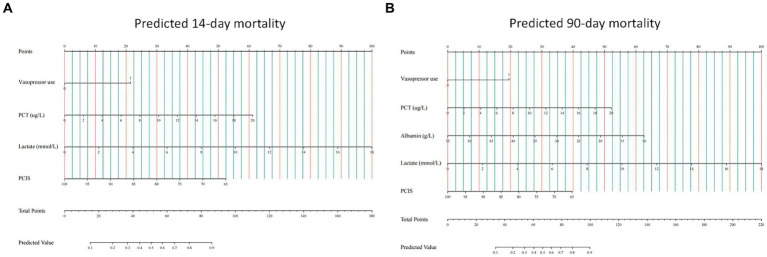
Construction of nomograms predicting 14-day mortality **(A)** and 90-day mortality **(B)** in the training cohort. The points of all features are added to obtain the total point, and a vertical line is drawn on the total point to obtain the corresponding ‘predicted value’, which indicates the risk of outcomes. PCT, procalcitonin; PCIS, pediatric critical illness score.

**Figure 5:**
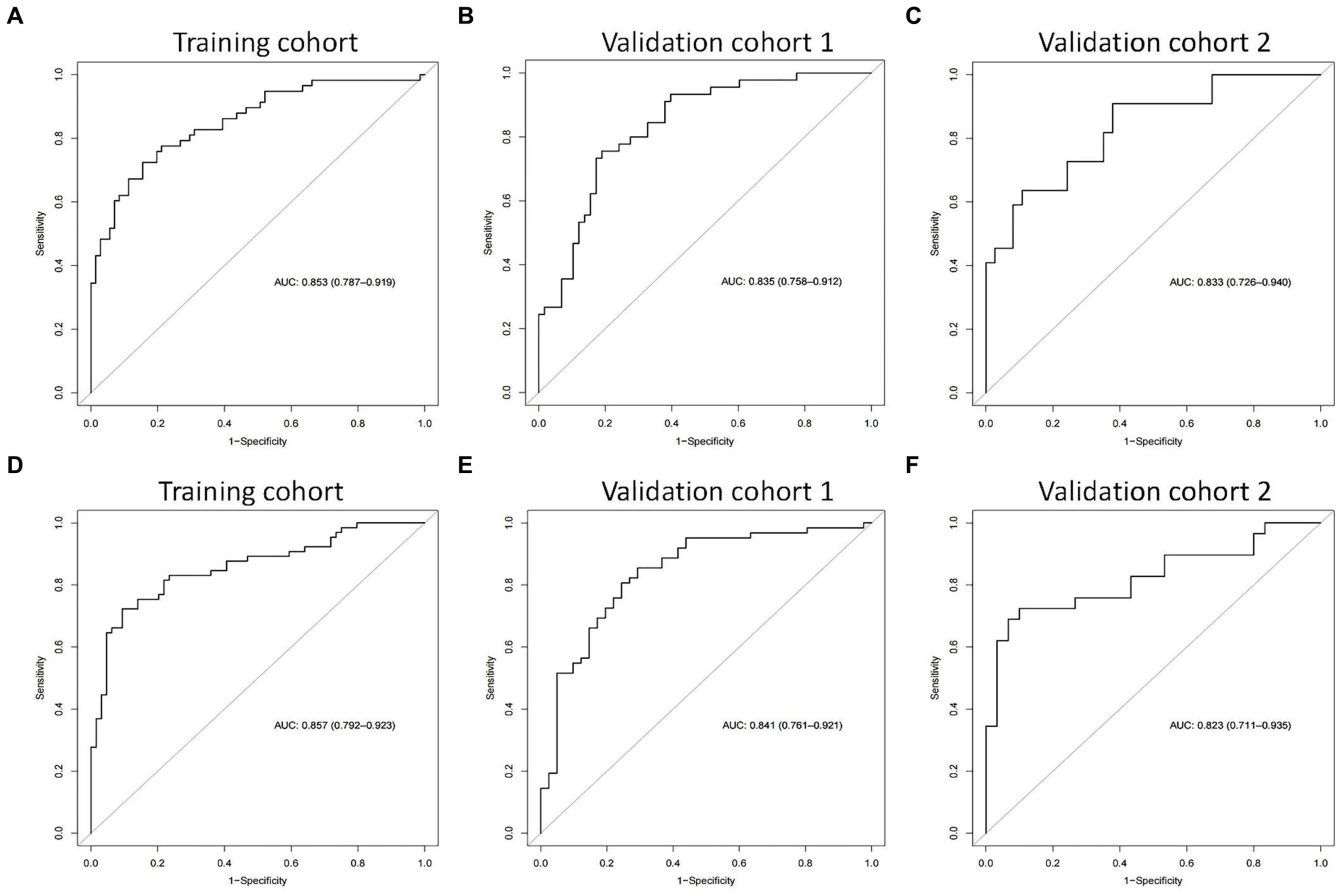
The receiver operating characteristic (ROC) curves of the nomograms in the training cohort, validation cohort 1, and validation cohort 2 for predicting 14-day mortality **(A–C)** and 90-day mortality **(D–F)**.

**Figure 6:**
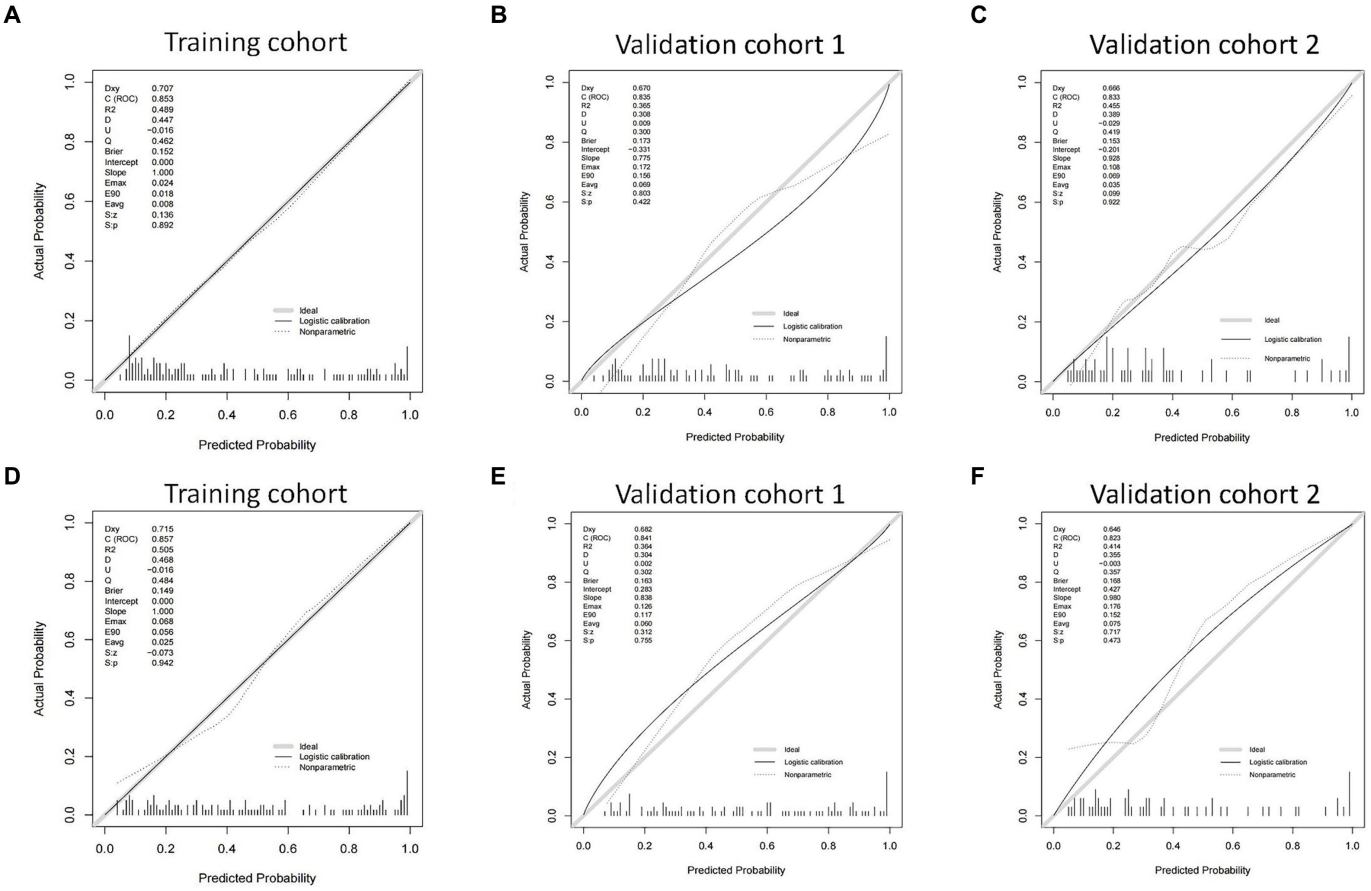
The calibration curves of the nomograms in the training cohort, validation cohort 1, and validation cohort 2 for predicting 14-day mortality **(A–C)** and 90-day mortality **(D–F)**.

**Figure 7:**
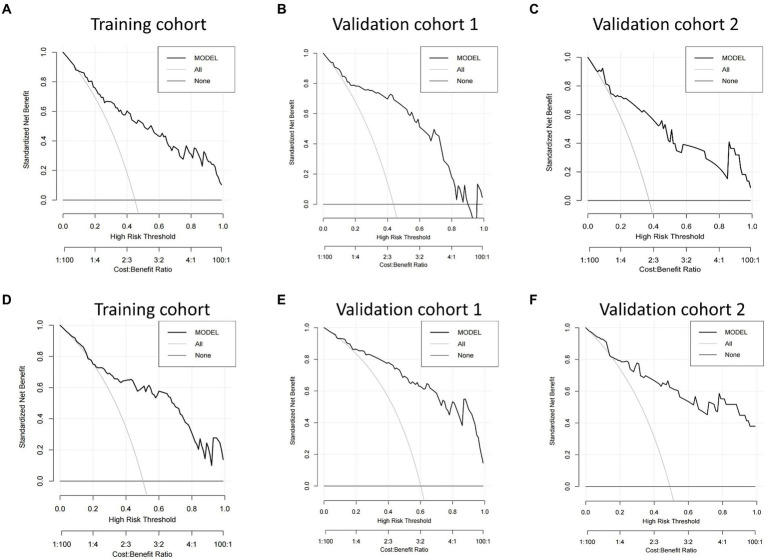
Decision curve analysis (DCA) of the nomograms. DCA compares the net benefits of three scenarios in predicting the risk of outcome: a perfect prediction model (gray line), screen none (horizontal solid black line), and screen based on the nomograms (ride line). The DCA curves were depicted in the training cohort, validation cohort 1, and validation cohort 2 for predicting 14-day mortality **(A–C)** and 90-day mortality **(D–F)**, respectively.

We then built nomogram models based on the logistic regression to predict 14-day mortality (Figure 4A) and 90-day mortality (Figure 4B). The performance of the models was identified by receiver operating characteristic (ROC) curves. In the training cohort, the AUC calculated by the bootstrap self-sampling method was 0.853 (95% CI 0.787–0.919) with a sensitivity of 72.4% and specificity of 84.5% for 14-day mortality (Figure 5A) and 0.857 (95% CI 0.792–0.923) with a sensitivity of 72.3% and specificity of 90.6% for 90-day mortality (Figure 5D), indicating high predictive power. Additionally, the calibration curve (Figures 6A,D) and decision curve (Figures 7A,D) revealed that the nomogram exhibited a good predictive ability for 14-day mortality and 90-day mortality, respectively.

The performance of the models was also assessed in validation cohorts. In validation cohort 1, the AUC was 0.835 (95% CI 0.758–0.912) with a sensitivity of 75.6% and specificity of 81.0% for 14-day mortality (Figure 5B) and 0.841 (95% CI 0.761–0.921) with a sensitivity of 80.6% and specificity of 75.6% for 90-day mortality (Figure 5E). In validation cohort 2, the AUC was 0.833 (95% CI 0.726–0.940) for patients with a sensitivity of 63.6% and specificity of 86.5% for 14-day mortality (Figure 5C) and 0.823 (95% CI 0.711–0.935) with a sensitivity of 75.9% and specificity of 80.0% for 90-day mortality (Figure 5F). The calibration curves also presented high agreement between prediction and observation in the risk of mortality in the two validation cohorts (Figures 6B,C,E,F).

### 3.4 Clinical application of the nomogram

DCA based on the net benefit and threshold probabilities was performed to test the clinical applicability of the nomogram models. Figure 7 shows that the risk prediction nomograms had a superior net benefit with a wide range of threshold probabilities in both the training cohort and validation cohorts.

### 3.5 Development of the online calculator

**Figure 8:**
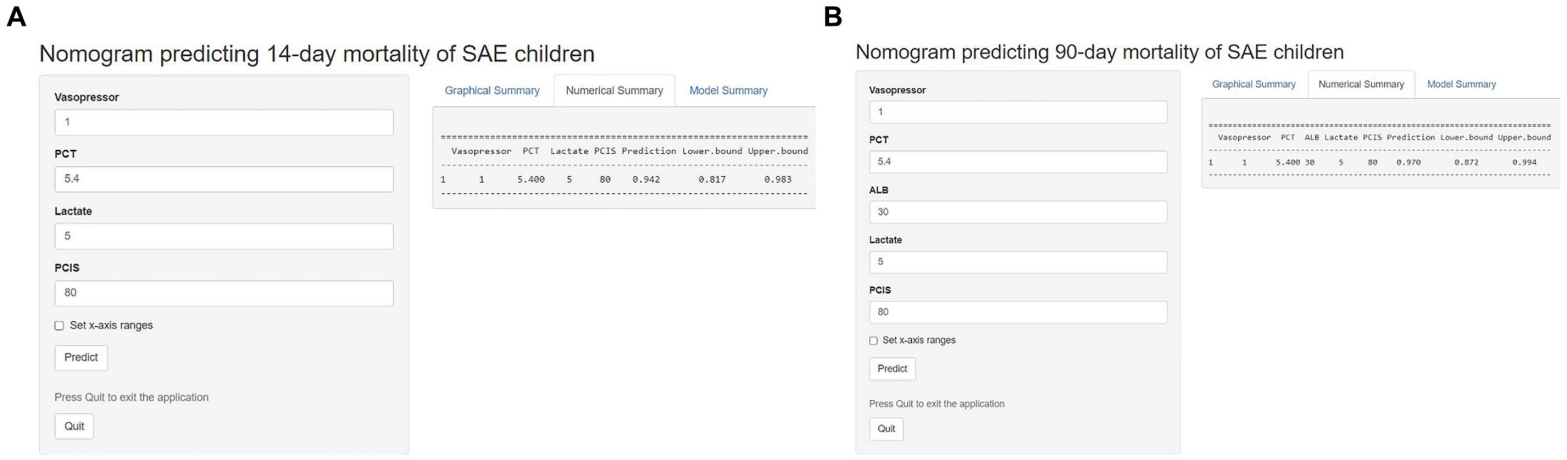
Two Internet browser-based online calculators for nomograms predicting 14-day mortality (website: https://johny2020.shinyapps.io/Dyn_14_mortality/) and 90-day mortality (website: https://johny2020.shinyapps.io/Dyn_90_mortality/).

Based on the nomogram models, two Internet browser-based online calculators were developed (Figure 8). After the user inputs the requested information of specific items, the probability of 14-day mortality^2^ or 90-day mortality^3^ in patients with SAE can be estimated.

## 4 Discussion

Our study revealed several key factors affecting the short- and long-term mortality of SAE, such as lactate, PCT, albumin, PCIS, and the use of vasopressors. These predictors were analyzed and integrated into the most suitable prediction model visualized as a prediction nomogram. Based on the external validation, our model showed relatively high AUC, sensitivity and specificity, indicating good reliability and discrimination. Our study is the first to investigate independent risk factors associated with mortality in SAE patients in the PICU across multiple centers.

Lactate is a critical prognostic indicator of sepsis in children (13, 14). A prospective cohort study among children with sepsis found that lactate levels within 4 h after admission were strongly associated with the occurrence of persistent organ dysfunction (15). We found that there was increased mortality within 90 days after PICU admission in SAE patients with higher lactate levels. Mechanistically, elevated serum lactate levels indicate microcirculatory dysfunction in patients, which results in tissue ischemia and hypoxia (16). Insufficient oxygen supply and inadequate nutrient delivery to the affected tissues may have severe consequences, particularly if vital organs such as the CNS are involved. Furthermore, prolonged insufficiency of oxygen supply leads to irreversible neuronal cell death and even permanent neurological disability, ultimately contributing to the occurrence of SAE (17). Overall, the measurement of lactate serves as a critical tool in assessing disease severity and predicting outcomes in pediatric sepsis and SAE patients.

As a calcitonin propeptidyl, serum PCT is elevated during the early stage of inflammation and infection and is thus always used as an early diagnostic test for infection (18). In fact, sepsis triggers a significant surge of proinflammatory mediators and leads to the disruption of anti-inflammatory defense in the body. The presence of these inflammatory factors, such as cytokines and chemokines, may disrupt the delicate balance of the body’s immunity mechanism, thereby exacerbating inflammation and causing subsequent tissue damage. Meanwhile, inflammatory mediators also have a direct impact on cerebral function by modulating cerebral blood flow and oxygen delivery, which constitutes the underlying mechanism of SAE (19, 20). Moreover, increasing evidence indicates that the surge of inflammatory factors during sepsis can compromise the integrity of the blood–brain barrier (BBB), leading to brain edema and brain injury, which is also the potential pathogenesis of SAE (19). In our study, PCT showed good predictive value for the mortality of SAE patients and was included in the nomogram model. Therefore, for patients with high levels of PCT, physicians should provide timely anti-infection and anti-inflammation therapy to reduce the mortality of SAE.

Our study demonstrated that the use of vasopressors was significantly higher in the nonsurvivors and was an independent risk factor for SAE death. For sepsis, a cascade of macrocirculatory and microcirculatory alterations may result in impaired vasoconstriction regulation, ultimately leading to severe hypotension (21). When sepsis-induced hypotension is refractory, the prognosis of septic shock is exceedingly poor. Impaired perfusion leads to dysfunction or failure of multiple vital organs, including the kidney, lung, liver, brain and heart (22), which may be associated with the development of SAE. The primary cause of death is typically attributed to resistant hypotension that fails to respond to volume resuscitation or to a high dose of vasopressors (23, 24). Moreover, there has been a shift in the preferred drug choice for fluid-refractory SAE patients from dopamine to either epinephrine or norepinephrine (25). Indeed, two randomized controlled trials comparing epinephrine and dopamine for SAE patients demonstrated that epinephrine yielded a better outcome (25). In conclusion, by identifying this independent risk factor, it may be possible to make more informed decisions about medication management.

At present, the commonly used pediatric critical scores include the PCIS, Pediatric Logistic Organ Dysfunction Score 2 (PELOD-2), Pediatric Multiple Organ Dysfunction Score (P-MODS) and Pediatric Early Warning Score (PEWS), among which the PCIS is extensively employed in China to assess the severity and prognosis of critically ill children (26). The comprehensive evaluation indicators included in the PCIS system are all objective variables obtained from laboratory examinations and physical examinations. The first PCIS after PICU admission can accurately reflect the severity of children’s condition and has certain value in judging the prognosis of diseases. Fang et al. (27) proved that the PCIS was negatively correlated with the number of combined systemic injuries for patients with severe *Mycoplasma pneumoniae* pneumonia, suggesting that a lower PCIS implied a higher possibility of multiple organ dysfunction. Our study confirmed that the PCISs were significantly decreased in nonsurviving patients compared with the surviving group, further supporting the accuracy of the PCIS as an independent predictor for 14-day and 90-day mortality in SAE patients.

Our research found that albumin occupies a substantial weight in the nomogram, indicating that it is also an important variable, particularly for predicting 90-day mortality in children with SAE. Previous studies have suggested that there is a correlation between serum albumin and the prognosis of patients with sepsis (28), but few studies have found a correlation between albumin and SAE. Higher albumin levels have been reported to be associated with a lower risk of delirium in patients with sepsis (29, 30), which supports our conclusion. Recently, many studies have demonstrated that in addition to being an indicator of malnutrition, there also exists a robust association between albumin and severe infection (31, 32). Low levels of albumin are prevalent among patients with severe sepsis and are associated with more severe inflammation (33–35). Reduced serum albumin levels predispose patients to brain edema, thereby leading to impaired consciousness and SAE (29, 30). Overall, albumin serves not only as an important prognostic marker but also as a potential therapeutic target for improving the outcome of critically severe sepsis and SAE patients. Appropriate patient evaluation followed by early initiation of enteral nutrition may be imperative for mitigating the risk of infection and organ dysfunction, thereby potentially reducing the incidence of SAE (30).

Recently, an increasing amount of evidence has suggested that PLT plays a crucial role not only in hemostasis and thrombosis but also in other various physiological processes, such as microbe-induced host defense, inflammatory processes, wound healing and tissue remodeling (36–38). Our study demonstrated a significant difference in PLT values between the deceased and surviving groups. The decreased PLT count observed in nonsurvivors may be related to the depletion of clotting factors and platelets during sepsis (39). Once platelets and coagulation factors are depleted, the formation of plasmin is increased, and then disseminated intravascular coagulation (DIC), a life-threatening syndrome, occurs. Therefore, early identification of thrombocytopenia and optimal management of these patients are essential keys to improving prognosis.

There are some limitations to this study that must be acknowledged. First, this is a retrospective analysis of the recorded data, and prospective studies are needed to identify the value of the nomogram in predicting mortality of SAE. Retrospective analyses rely on the existing data and may not provide a comprehensive understanding of all factors influencing the outcomes. Additionally, as this study was limited to PICU patients, the generalizability to other populations may be constrained. Although the developed models have already been validated in two hospitals, external validation across diverse regions is still necessary. Thus, further research involving diverse populations from various health care settings would enhance the applicability and validity of the findings.

## 5 Conclusion

A nomogram was established for predicting the 14-day and 90-day mortality of SAE patients admitted to the PICU, which served as a valuable tool in the medical field. After multicenter external validation, the nomogram showed good reliability and discrimination. It will be particularly beneficial in preventing SAE deterioration and ultimately improving the prognosis of children with SAE.

## Data availability statement

The original contributions presented in the study are included in the article/supplementary material, further inquiries can be directed to the corresponding author.

## Ethics statement

The studies involving humans were approved the Institutional Ethics Review Board of Qilu Hospital of Shandong University (Approval number: KYLL-202202-027-1). The studies were conducted in accordance with the local legislation and institutional requirements. Written informed consent for participation was not required from the participants or the participants' legal guardians/next of kin in accordance with the national legislation and institutional requirements.

## Author contributions

GW: Writing – original draft, Writing – review & editing. YG: Writing – original draft, Writing – review & editing. YF: Data curation, Writing – original draft. QH: Data curation, Writing – original draft. EG: Data curation, Writing – original draft. QJ: Data curation, Writing – original draft. JL: Data curation, Writing – original draft. XJ: Data curation, Writing – original draft. XL: Writing – review & editing.

## Footnotes

^1^ https://www.r-project.org

^2^ https://johny2020.shinyapps.io/Dyn_14_mortality/

^3^ https://johny2020.shinyapps.io/Dyn_90_mortality/
